# Supplementary material for: The burden of medical contraindications to corneal donation: Time for review
Source: PLOS Glob Public Health. 2024 Dec 31;4(12):e0003537. doi: 10.1371/journal.pgph.0003537 (PMC11687701; doi:10.1371/journal.pgph.0003537)
Supplement: S1 Table — These recommendations comply with the selection criteria applicable to tissue donors as set out in Annex I of European Directive EC 2006/17 and Annex II of the Order of November 4, 2014. In red are those we question. (DOCX) [file pgph.0003537.s001.docx]

**S1 Table.** Comprehensive list of **contraindications to corneal donation, classified by major category.** These recommendations comply with the selection criteria applicable to tissue donors as set out in Annex I of European Directive EC 2006/17 and Annex II of the Order of November 4, 2014. In red are those we question.

| **Cancer risk** | **Exceptions for corneal donation** |
| --- | --- |
| Hematological malignancies |  |
| Myelodysplastic syndrome |  |
| Leukemia |  |
| Myeloproliferative syndrome: Vaquez disease  Chronic myeloid leukemia  Essential thrombocythemia Uninvestigated polycythemia |  |
| Monoclonal gammopathy of undetermined significance (MGUS) |  |
| Myeloma |  |
| Lymphoma |  |
| Medullar aplasia |  |
| Unlabelled macrophagic activation syndrome |  |
| Myelemia >2% not investigated |  |
| Cancers affecting the eye |  |
| Melanoma |  |
| Malignant tumors of the central nervous system |  |
| Neoplastic meningitis and lymphangitic carcinomatosis | No other cancer, whatever its stage, is a contraindication. |
| **Risk of transmission of prion diseases =**  Transmissible Spongiform Encephalopathies (TSE) |  |
| -Creutzfeldt-Jakob disease (all variants)  -Gertsmann Strausser Scheinker syndrome (GSS)  -Fatal familial insomnia (FFI)  -Kuru  -Family history of genetic prion disease |  |
| People with a history of rapidly progressing dementia:  Continuous cognitive impairment for less than 2 years |  |
| People with a history of degenerative neurological diseases, including those of unknown origin:  -Alzheimer's disease  -Parkinson's disease and Parkinsonian syndromes  -Amyotrophic lateral sclerosis  -Multisystem atrophy (MSA)  -Huntington's disease  -Lewy body dementia  -Pick's disease  -Fronto-parietal dementia  -Congenital Rubella  -Progressive multifocal leukoencephalitis | -Dementia of identified origin (e.g.: dementia of vascular origin evolving for more than 2 years and diagnosed as such by a neurologist)  Guillain-Barré syndrome treated and cured  -Demyelinating or chronic polyradiculoneuritis  -Progressive spinal muscular atrophy  -Myopathy |
| -Subacute sclerosing encephalitis  -Reye's syndrome  -Strümpell-Lorrain disease (hereditary spastic paraplegia)  -Devic's disease  Tropical spastic paraparesis (HTLV-1- associated myelopathy)  -Amyloid angiopathy |  |
| -Treatment with extractive pituitary hormones before 1989  -Treatment with placental glucocerebrosidase (Gaucher disease) |  |
| Cornea, sclera and dura mater allografts |  |
| Neurosurgical procedure, excluding bone spine surgery, before 2001 and for which dura mater of human origin may have been used, involving:  Brain, spinal cord, olfactory and optic nerves, retina, olfactory mucosa, inner ear |  |
| Trip or stay of more than one year/6 months  cumulated in Great Britain between 1980 and 1996 |  |
|  |  |
| **Risk of hemodilution**  >50% of plasma volume or exchange of ½ blood mass |  |
|  |  |
| **Risk of infection** |  |
| Uncontrolled systemic infection at the time of donation:  -Systemic mycosis  -Active viral infection  HIV, Hepatitis ABC, Active malignant influenza, Active CMV, Syphilis, Zika, West Nile, Chicungunya, Dengue, Varicella, Shingles. | Bacterial sepsis in donated corneas for organoculture storage |
| Unexplained fever | Malaria cured |
| Local infection contraindicating donation | Tuberculosis cured |
| -History, risk factors and corresponding physical signs, clinical evidence or test results + for HIV, HBV, HCV and/or HTLV  -History of intravenous drug use, risky sexual behavior, prison stay | Profile of formerly cured hepatitis B |
| Previous vaccination with live attenuated virus less than 4 weeks old |  |
| Returning from a trip to an endemic epidemic zone |  |
|  |  |
| **Specific contraindication for cornea** |  |
| Retinoblastoma, malignant anterior chamber tumor |  |
| Eye infection or inflammation |  |
| Ophthalmic Zona (current and past) |  |
| Herpes |  |
| Lamellar implants, or anterior chamber implant, radial  Keratotomy |  |
| Corneal opacity, scars, pterygium or other superficial disorders of the conjunctiva or cornea only if located  in the visual axis | Not applicable for endothelial keratoplasty |
| Gerontoxon only if clear zone diameter compromised (<7mm) | Not applicable for endothelial keratoplasty |
| Behcet's disease |  |
| Gougerot Sjögren's syndrome | Not applicable for endothelial keratoplasty |
| Lyell syndrome |  |
| Marfan syndrome | Not applicable for endothelial keratoplasty |
