# Supplementary material for: The burden of medical contraindications to corneal donation: Time for review
Source: PLOS Glob Public Health. 2024 Dec 31;4(12):e0003537. doi: 10.1371/journal.pgph.0003537 (PMC11687701; doi:10.1371/journal.pgph.0003537)
Supplement: Attachment — Submitted filename: ResponsetoReviewers.docx [file pgph.0003537.s003.docx]

Saint-Etienne and Melbourne, September 14th, 2024

Dear Editor, dear M. Cowley,

Please find enclosed our revised manuscript entitled " The burden of medical contraindications to corneal donation: time for review

.

We would like to thank the reviewer for helpful criticisms and recommendations to strengthen the paper.

The revised manuscript responds fully and directly to the comments of the reviewers. As per your instructions, all amendments are specified in our point-by-point response and marked blue in the revised manuscript.

We sincerely hope this revised version of the manuscript will meet the reviewers’ requirements and that you will find it suitable for publication in PLOS Global Public Health

Yours sincerely, on behalf of all authors,

**Prof. Gilles Thuret**

*Saint-Etienne University Hospital*

*Ophthalmology Department*

*Laboratory of Biology, Engineering, and Imaging for Ophthalmology, BiiO ,*

*Faculty of Medicine*

*Saint-Etienne*

*France*

CC: hcowley@plos.org

PGPH-D-24-01468
The burden of medical contraindications to corneal donation: time for review
PLOS Global Public Health

Dear Dr. Thuret,

Thank you for submitting your manuscript to PLOS Global Public Health. After careful consideration, we feel that it has merit but does not fully meet PLOS Global Public Health’s publication criteria as it currently stands. Therefore, we invite you to submit a revised version of the manuscript that addresses the points raised during the review process.

Please note that we have only been able to secure a single reviewer to assess your manuscript. We are issuing a decision on your manuscript at this point to prevent further delays in the evaluation of your manuscript. Please be aware that the editor who handles your revised manuscript might find it necessary to invite additional reviewers to assess this work once the revised manuscript is submitted. However, we will aim to proceed on the basis of this single review if possible.

The reviewer has commented on several aspects of your manuscript, and has provided some suggestions on how these can be addressed. Please ensure you address each of the reviewer's comments when revising your manuscript.

Please submit your revised manuscript by Oct 21 2024 11:59PM. If you will need more time than this to complete your revisions, please reply to this message or contact the journal office at globalpubhealth@plos.org. When you're ready to submit your revision, log on to https://www.editorialmanager.com/pgph/ and select the 'Submissions Needing Revision' folder to locate your manuscript file.

Please include the following items when submitting your revised manuscript:

- A rebuttal letter that responds to each point raised by the editor and reviewer(s). You should upload this letter as a separate file labeled 'Response to Reviewers'.
- A marked-up copy of your manuscript that highlights changes made to the original version. You should upload this as a separate file labeled 'Revised Manuscript with Track Changes'.
- An unmarked version of your revised paper without tracked changes. You should upload this as a separate file labeled 'Manuscript'.

Guidelines for resubmitting your figure files are available below the reviewer comments at the end of this letter.

We look forward to receiving your revised manuscript.

Kind regards,

Hugh Cowley

Staff Editor

PLOS Global Public Health

Journal Requirements:

Additional Editor Comments (if provided):

[Note: HTML markup is below. Please do not edit.]

Reviewers' comments:

Reviewer's Responses to Questions

**Comments to the Author**

1. Does this manuscript meet PLOS Global Public Health’s [publication criteria](https://c05y1x9s.r.us-east-2.awstrack.me/L0/https:%2F%2Fjournals.plos.org%2Fglobalpublichealth%2Fs%2Fcriteria-for-publication/1/010f0191d5aac6a0-e36c85c1-0616-414a-82c7-dfb1a149fa16-000000/zYF1vlcK7MMa-i3QQYJ_Bc00n0o=175)? Is the manuscript technically sound, and do the data support the conclusions? The manuscript must describe methodologically and ethically rigorous research with conclusions that are appropriately drawn based on the data presented.

Reviewer #1: Yes

2. Has the statistical analysis been performed appropriately and rigorously?

Reviewer #1: Yes

3. Have the authors made all data underlying the findings in their manuscript fully available (please refer to the Data Availability Statement at the start of the manuscript PDF file)?

The [PLOS Data policy](https://c05y1x9s.r.us-east-2.awstrack.me/L0/https:%2F%2Fjournals.plos.org%2Fglobalpublichealth%2Fs%2Fdata-availability/1/010f0191d5aac6a0-e36c85c1-0616-414a-82c7-dfb1a149fa16-000000/fp5PDxaDzsv2uGBfkcl10pA65Uw=175) requires authors to make all data underlying the findings described in their manuscript fully available without restriction, with rare exception. The data should be provided as part of the manuscript or its supporting information, or deposited to a public repository. For example, in addition to summary statistics, the data points behind means, medians and variance measures should be available. If there are restrictions on publicly sharing data—e.g. participant privacy or use of data from a third party—those must be specified.

Reviewer #1: Yes

4. Is the manuscript presented in an intelligible fashion and written in standard English?

PLOS Global Public Health does not copyedit accepted manuscripts, so the language in submitted articles must be clear, correct, and unambiguous. Any typographical or grammatical errors should be corrected at revision, so please note any specific errors here.

Reviewer #1: Yes

5. Review Comments to the Author
Please use the space provided to explain your answers to the questions above. You may also include additional comments for the author, including concerns about dual publication, research ethics, or publication ethics. (Please upload your review as an attachment if it exceeds 20,000 characters)

Reviewer #1: The authors present a compelling argument that there is sufficient evidence to revisit regulatory restrictions on eye donation that were instituted long ago. These restrictions may be creating artificial barriers to treatment and cornea accessibility with little to no increase in recipient safety. This paper raises important questions for the eye banking and corneal transplant community. I believe, with minor revisions, this paper should be published.

Line 155. This line is a little awkward. Is the procedure “a view to donation” an approach of the next of kin for donation authorization?

Indeed! We have corrected according to your recommendations.

Carcinomatous meningitis and lymphangitis are not commonly used terms in the US. I presume others might wish to have these diseases defined so that a broader audience can understand the CIs described a little better.

We suggest to replace these terms by “neoplastic meningitis and lymphangitic carcinomatosis” which are the most cited terms in PubMed. We added a brief description of both: end-stage carcinomatous infiltration respectively of the meninges and of the lymphatic vessels, usually pulmonary

DISCUSSION

Your results might be different without COVID. That said, the impact on the additional donors potentially suitable for transplant would not change. For example, 53% of the CI are part of the five Diseases (5D) you posit should be reconsidered. Without COVID as a significant cause of death in your donor pool, 5D would account for a higher percentage of ruled out donors.

That's right, and it's one of the reasons why we decided to publish our data, even though some of it was collected during the CIVID-19 pandemic. Finally, the impact of these 5 groups of diseases remains enormous, whether or not there is another frequent but fortunately temporary medical contraindication such as Covid!

Line 235. Eye Banking of America should be “Eye Bank Association of America”.

OK, done.

Line 274-304. Arguments for differentiating between CJD and Alzheimer disease must rely on observable clinical manifestations of the diseases. You do address this later in the section, but I think you can state this more succinctly. I recommend removing some of the discussion on the basic science related to the brain pathology.

We removed the “basic science” part accordingly

Line 286. Please ensure that there is a space between that and normal.

OK, done.

Line 305. You discuss melanoma transmission as related to KLAL, but not breast CA which was reported in this paper: <https://pubmed.ncbi.nlm.nih.gov/28476053/>

Thank you for your comment. We were aware of this publication but had forgotten to mention it. We've added it... in the hope that it won't inspire some zealous authority to add a general contraindication on breast cancer!!!!

Line 313-317. I feel like this is an odd comparison. With Allopurinol, there may not be other alternative therapies.

Our idea was to deliberately take a comparison from the everyday life of doctors and patients. There are alternatives to allopurinol (other xanthine oxidase inhibitors, Pegloticase, probenecid), yet over 10 million boxes are sold in France every year (<https://uspo.fr/wp-content/uploads/2014/06/ANSM_Analyse-Ventes-Medicaments_2013.pdf>). Respectfully, we would like to keep it. Let’s see how readers will react!

With cornea transplant, we could do a number of things to increase tissue availability without adjusting the CIs. For example, in the US, we discard many thousands of corneas due to hepatitis B core antibody testing which is required by the FDA. The risk from these corneas is negligible. But tell that to the FDA when they know we have access to more corneas available in the US than needed in the US. Negligible risks become unacceptable risks. This could be factored into the rationale of the French mindset. Risk assessment is likely based on local need and not on global demand.

This is so interesting! In Europe and Australia, serology profiles of formerly cured hepatitis B is not a contra-indication.

You are totally right about the risk interpretation according to the local need by health authorities. The fact is that in France (and perhaps in Europe too) we will never reach the wonderful efficacy of the American system for corneal procurement. Our dream, as ophthalmologist, would be that Europe significantly contribute to export corneas as efficiently as USA does! But we are very very far from that!

Line 327. Add “to” before “determine”

OK, done.

One item I didn’t see mentioned in the discussion is the inefficiency and waste incurred by the system when CIs are introduced. Our staff spends a lot of time researching disease states to ensure that we don’t inadvertently transplant tissue that is unsuitable based on our regulations. The fewer diseases to worry about, the easier it is to ensure that the diseases that really do pose a risk to recipients are not present. Unfortunately, we find things in the record after the fact that rule recovered tissue ineligible and this adds a lot of burden to the health care system and it is unfortunate when this happens for the donor and the family that gave permission to proceed. Additionally, people who want to donate tissues as an altruistic act may be excluded by these rules – we should not be creating artificial barriers to do good in the world. Only address these items in your discussion if you feel they would add to your paper.

We couldn't agree with you more. We'd like to add the following notions to the discussion: 1/the risk of dispersal for coordination teams; 2/the stigmatization of donors and their families.

We suggest:

In our opinion, the excessive number of medical contraindications to cornea donation has at least 2 other harmful consequences: 1/ it needlessly overloads the work of the coordination teams. A reasoned reduction of this list would enable them to focus on the search for truly dangerous diseases. In this respect, our study clearly demonstrates that signs of CJD are drowned out by the “cognitive decline” item; 2/ it unnecessarily stigmatizes these donors and their relatives, who are denied the opportunity to generously donate.

SUPPLEMENT

Is listing herpes really absurd if it is noted in the corneal history? I would exclude that tissue. You are correct that it would be rare to have this history (or access to it), but it is not as rare as some of the other diseases noted. This comment should probably be removed as it doesn’t really advance your thesis.

Thank you for this discussion, which confirms that we misspoke. It is not illogical to leave a history of ocular herpes in the list of contraindications. However, as this is very often an ancient history, very often absent from the general medical record and forgotten by families, this search is absolutely not “sensitive”, nor specific for that matter! 
If the group of experts behind this IC had been logical, they would have made it compulsory to test for HSV1, HSV2 and VZV DNA in the graft storage medium...
We are deleting this comment in accordance with your recommendations.

6. PLOS authors have the option to publish the peer review history of their article ([what does this mean?](https://c05y1x9s.r.us-east-2.awstrack.me/L0/https:%2F%2Fjournals.plos.org%2Fglobalpublichealth%2Fs%2Feditorial-and-peer-review-process%23loc-peer-review-history/1/010f0191d5aac6a0-e36c85c1-0616-414a-82c7-dfb1a149fa16-000000/sPHZ7qF7R9bwefZtQTQiVsL6jgg=175)). If published, this will include your full peer review and any attached files.

**Do you want your identity to be public for this peer review?** If you choose “no”, your identity will remain anonymous but your review may still be made public.

For information about this choice, including consent withdrawal, please see our [Privacy Policy](https://c05y1x9s.r.us-east-2.awstrack.me/L0/https:%2F%2Fwww.plos.org%2Fprivacy-policy/1/010f0191d5aac6a0-e36c85c1-0616-414a-82c7-dfb1a149fa16-000000/7IQYnAzG86dUdtIIKowCEPrQuvQ=175).

Reviewer #1: No

[NOTE: If reviewer comments were submitted as an attachment file, they will be attached to this email and accessible via the submission site. Please log into your account, locate the manuscript record, and check for the action link "View Attachments". If this link does not appear, there are no attachment files.]

While revising your submission, please upload your figure files to the Preflight Analysis and Conversion Engine (PACE) digital diagnostic tool, https://pacev2.apexcovantage.com/. PACE helps ensure that figures meet PLOS requirements. To use PACE, you must first register as a user. Registration is free. Then, login and navigate to the UPLOAD tab, where you will find detailed instructions on how to use the tool. If you encounter any issues or have any questions when using PACE, please email PLOS at figures@plos.org. Please note that Supporting Information files do not need this step.
