# Supplementary material for: The burden of medical contraindications to corneal donation: Time for review
Source: PLOS Glob Public Health. 2024 Dec 31;4(12):e0003537. doi: 10.1371/journal.pgph.0003537 (PMC11687701; doi:10.1371/journal.pgph.0003537)
Supplement: Attachment — Submitted filename: ResponsetotheReviewers.docx [file pgph.0003537.s004.docx]

Saint-Etienne and Melbourne, November 1^st^, 2024

Dear Editor, dear M. Russel,

Please find enclosed our revised manuscript entitled " The burden of medical contraindications to corneal donation: time for review

.

We would like to thank the reviewer for recommendations to strengthen the paper.

The revised manuscript responds fully and directly to the comments of the reviewers. As per your instructions, all amendments are specified in our point-by-point response and marked red in the revised manuscript.

We sincerely hope this revised version of the manuscript will meet the reviewers’ requirements and that you will find it suitable for publication in PLOS Global Public Health

Yours sincerely, on behalf of all authors,

**Prof. Gilles Thuret**

*Saint-Etienne University Hospital*

*Ophthalmology Department*

*Laboratory of Biology, Engineering, and Imaging for Ophthalmology, BiiO ,*

*Faculty of Medicine*

*Saint-Etienne*

*France*

CC: alton.russell@mcgill.ca

PGPH-D-24-01468R1
The burden of medical contraindications to corneal donation: time for review PLOS Global Public Health Dear Dr. Thuret,

Thank you for submitting your manuscript to PLOS Global Public Health. After careful consideration, we feel that it has merit but does not fully meet PLOS Global Public Health’s publication criteria as it currently stands. Therefore, we invite you to submit a revised version of the manuscript that addresses the points raised during the review process.

Although Reviewer 1 indicated they were satisfied with your revision, I was newly assigned your paper as an academic editor and asked to review your paper in light of the fact that only one external peer reviewer had been identified. I agree that paper is suitable for publication in PLOS Global Public Health, but believe the manuscript can be strengthened by addressing my comments below.

Please submit your revised manuscript by Nov 08 2024 11:59PM. If you will need more time than this to complete your revisions, please reply to this message or contact the journal office at globalpubhealth@plos.org. When you're ready to submit your revision, log on to https://www.editorialmanager.com/pgph/ and select the 'Submissions Needing Revision' folder to locate your manuscript file.

Please include the following items when submitting your revised manuscript:

- A rebuttal letter that responds to each point raised by the editor and reviewer(s). You should upload this letter as a separate file labeled 'Response to Reviewers'.
- A marked-up copy of your manuscript that highlights changes made to the original version. You should upload this as a separate file labeled 'Revised Manuscript with Track Changes'.
- An unmarked version of your revised paper without tracked changes. You should upload this as a separate file labeled 'Manuscript'.

Guidelines for resubmitting your figure files are available below the reviewer comments at the end of this letter.

We look forward to receiving your revised manuscript.

Kind regards, W. Alton Russell, PhD

Academic Editor

PLOS Global Public Health

Journal Requirements:

Please review your reference list to ensure that it is complete and correct. If you have cited papers that have been retracted, please include the rationale for doing so in the manuscript text, or remove these references and replace them with relevant current references. Any changes to the reference list should be mentioned in the rebuttal letter that accompanies your revised manuscript. If you need to cite a retracted article, indicate the article’s retracted status in the References list and also include a citation and full reference for the retraction notice.

To the best of our knowledge, our reference list does not contain any articles that have been withdrawn.

Additional Editor Comments (if provided):

1.Please clarify your approach to classifying decedents with >1 contraindication. Did you capture all contraindications for each decedent, or only one? If only one was recorded, how was it chosen? If some of the patients who were contraindicated due to one of the conditions in your five groups had another contraindication, couldn't your estimate that removing these groups would increase the supply by 71% could be slightly overestimated?

Thank you for your question. Multiple CIs were not systematically looked for as this was of no interest in daily practice, since a single CI is sufficient to exclude the potential donor. We were therefore able to consider only one CI for the analysis, the one that the nurses noted first, and which stopped the donation procedure.

We have added this clarification to the text as follows L161: “*We have only taken into account one CI per person, since in practice, one was enough to stop the procedure*”

Given the absence of these data, unfortunately, we are unable to respond to this request.

2. For each table reporting n (%) please clarify in the table caption whether categories are mutually exclusive or if some individuals are counted in more than one category.

For all tables concerned, the categories were mutually exclusive. Each individual was counted only once. This is now specified in the 3 table legends

3. Supplemental Figure 1 is really a table, so should be called supplemental table 1

Absolutely!

4. A space is missing between words on lines 145 and 200

The space is there but the word layout seems to be faulty....

5. (optional) You might consider addressing reviewer 1’s comment on estimating the relative contribution of your five ‘disease groups in question’ quantitatively. You can quantify the proportion of Cis that would have been due to one of your five diseases groups without COVID, and you could estimate what the percent increase in donated corneas would have been if it weren’t for COVID.

Throughout the Covid period, other contraindications were not systematically investigated. It should be noted that throughout this period, coordination was focused on COVID, ‘forgetting’ everything else.

Unfortunately, we are unable to respond to this request.
